# Supplementary figures and images for: BMP pathway regulation of insulin signaling components promotes lipid storage in Caenorhabditis elegans
Source: PLoS Genet. 2021 Oct 11;17(10):e1009836. doi: 10.1371/journal.pgen.1009836 (PMC8530300; doi:10.1371/journal.pgen.1009836)

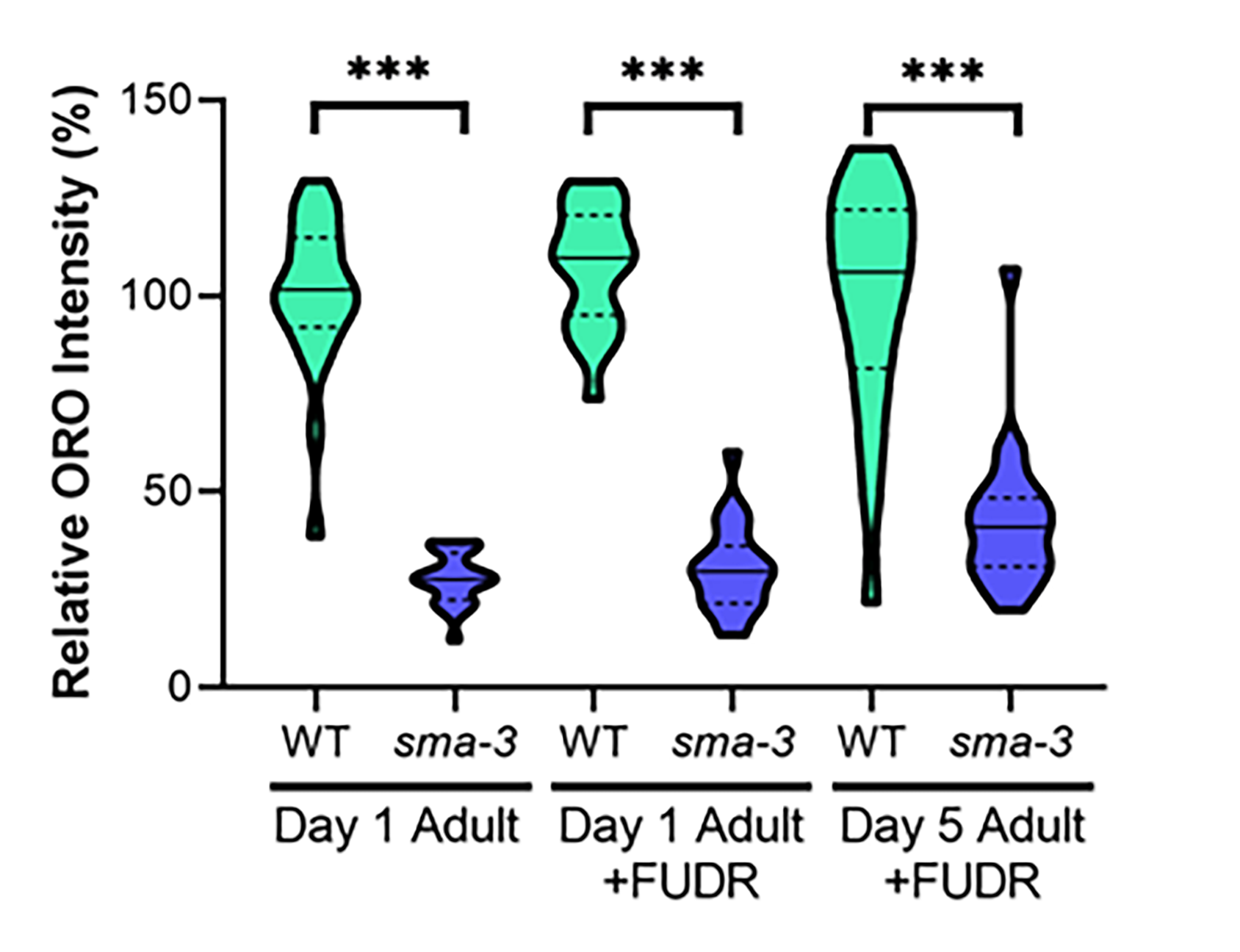

Supplement: S1 Fig — sma-3 animals exhibit reduced lipid accumulation during adulthood via ORO staining. This reduction is independent of reproduction, as treatment with FUDR prevents the development of eggs. These results suggest that sma-3 continues to affect lipid accumulation through development and into adulthood. Asterisks across the bottom denote significance compared to Control, n.s. not significant, * p value < 0.05, *** p value <0 .001, solid lines denote the median, dashed lines denote quartiles. (TIF) [file pgen.1009836.s001.tif]

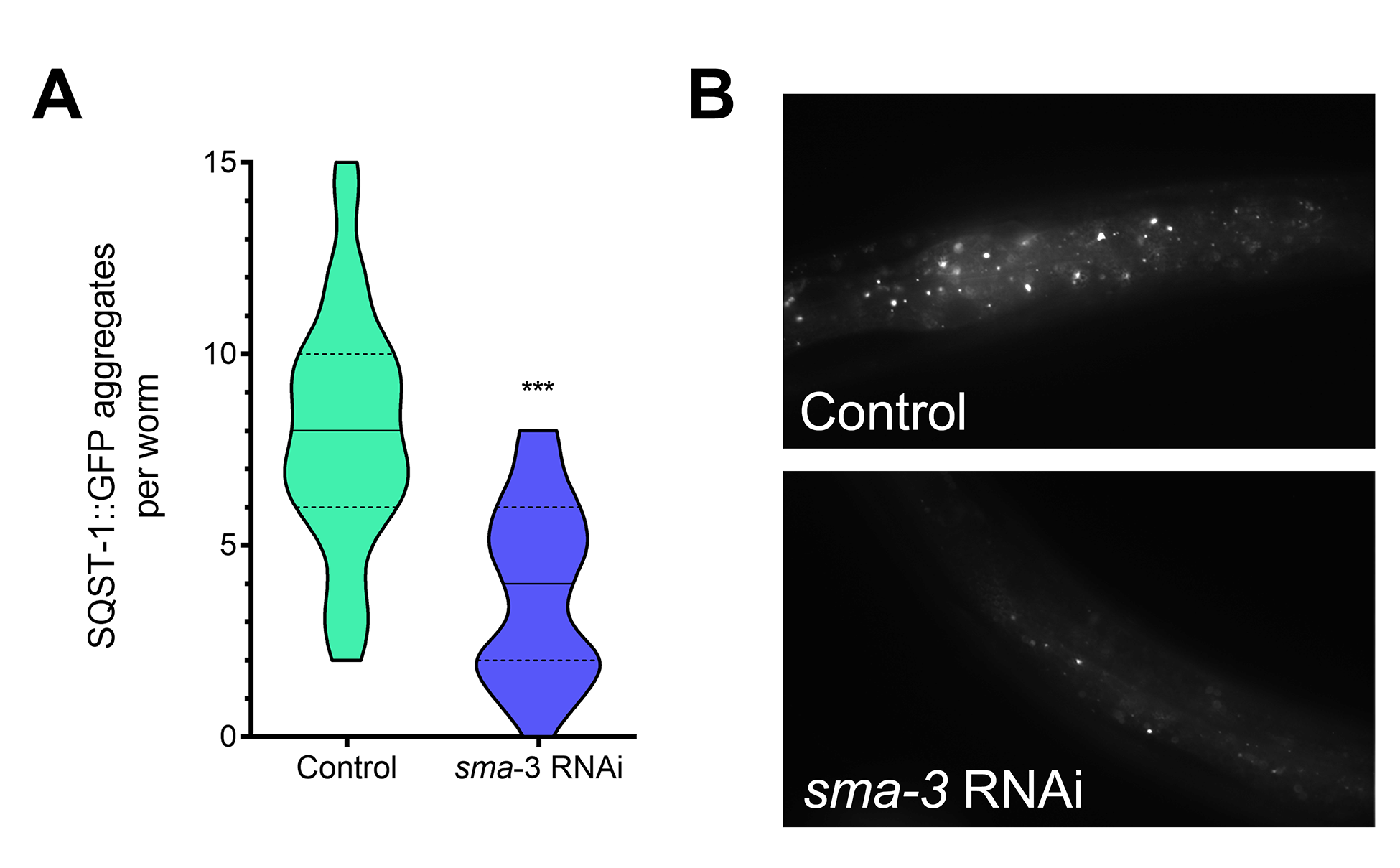

Supplement: S2 Fig — A) rpl-43(bp399) mutants show accumulation of SQST-1::GFP puncta due to impaired ribosomal activity [38]. Genetic backgrounds that increase autophagy allow the clearance of those aggregates. We analyzed rpl-43 mutants at the L4 stage following RNAi depletion of sma-3/Smad compared with empty vector control (L4440), and found a significant clearance of SQST-1::GFP puncta (p < 0.001) as expected [38]. Two trials of n = 15 animals yielded similar results, and the combined data are shown. B) Images of sqst-1::gfp animals treated with either an empty vector control (L4440) or sma-3 RNAi taken at 400X. (TIF) [file pgen.1009836.s002.tif]
