# Supplementary material for: BMP pathway regulation of insulin signaling components promotes lipid storage in Caenorhabditis elegans
Source: PLoS Genet. 2021 Oct 11;17(10):e1009836. doi: 10.1371/journal.pgen.1009836 (PMC8530300; doi:10.1371/journal.pgen.1009836)
Supplement: Attachment — Submitted filename: Response to Reviewers v2.docx [file pgen.1009836.s003.docx]

**Response to Reviewers**

We are submitting a revision of our manuscript “*BMP Pathway Regulation of Insulin Signaling Components to Promote Lipid Storage and Homeostasis*”. We thank the reviewers and the editors for their thoughtful reviews of our previous submission (PGENETICS-D-19-01656), based on which we have made substantial changes that strengthen the work significantly. The editorial comments noted that, although an important topic, the manuscript was not complete enough for PLOS Genetics. We have chosen to increase our focus on the previously unexplored relationship between insulin-like peptide INS-4 and downstream transcription factors DAF-16/FoxO and SKN-1/Nrf in lipid homeostasis. We performed new experiments and made text revisions in response to reviewers’ comments. In addition, when re-analyzing the *ins‑4;sma‑3* double mutant we discovered that contamination was responsible for the previously observed high-fat phenotype of this strain. Following decontamination by bleaching, the strain demonstrates a low-fat phenotype as described in the current version of the paper.

Summary of changes to Figures:

| **Current** | **Previous** |
| --- | --- |
| Figure 1A | Figure 1A |
| Figure 1B | new |
| Figure 1C | Figure 1B |
| Figure 2A,B | Figure 3A,B |
| Figure 2C,D | Figure 4A,B |
| Figure 3A | Figure 5 |
| Figure 3B,C | new |
| Figure 4A | new |
| Figure 4B,C | Figure 2A,B |
| Figure 5 | Figure 6 |
| S1 Fig | new |
| S2 Fig | S1 Fig |

In response to specific comments of the reviewers:

**Reviewer #1:**

1) *I think the most interesting and novel aspect of this paper is down-regulation of ins-4 by BMB signaling and the role of ins-4 in lipid metabolism. When I was reading the manuscript, I expected that they would build up this paper from here and would show some more genetic interaction among INS-4, BMP signaling and IIS in the context of lipid metabolism, dauer formation and autophagy*.

**We are grateful for reviewer 1’s suggestions and we have added new experiments in Figure 3B,C that show that the loss of *daf-16* blocks the high-fat phenotype of *ins-4* mutants and that DAF-16::GFP nuclear localization in *daf-2* RNAi animals is sensitive in *ins-4* overexpression.**

*2) In figure 1, it will be better to show that ins-4 mRNA levels are increased in sma-3 mutants using qRT-PCR and to quantify the fluorescence intensity to further support their conclusion.*

**We have added new qRT-PCR data in Figure 1B, where we quantify *ins-4* mRNA levels in *sma-3* mutants.**

*3) For figure 5 Oil Red O assay, it will be better to use daf-2 RNAi or daf-2(e1370) and ins-4 mutation in conjunction with daf-16 mutation (or RNAi), skn-1 mutation (or RNAi), and dbl-1 mutation.*
**We have performed these experiments with *ins-4* mutants (Figure 3B). Others have analyzed *daf-2* interactions with *daf-16* (*e.g.* Aghayeva et al., PLOS Biology 2021), and we previously published *daf-2;dbl-1* Oil Red O results (Clark et al., 2018).**

Minor comments
*1) The description about lon-2, a negative regulator of DBL-1 is missing in the Introduction but they used lon-2 mutants for their experiments. Please add the description about lon-2 in the Introduction and include citation, such as Gumienny et al., 2007, Current Biology.*
**We have added this change to the introduction (p. 4).**

*2) It will be better to choose another representative image of daf-2 RNAi-treated control worms in page 26, figure 3B and the representative images of “Med” and “Low” worms in page 28, figure 4A.*

**We consolidated figures 3 and 4 from the previous version and chose representative images for “High”, “Med”, and “Low”.**

*3) In Abstract, the authors wrote “The molecular components required for this interaction, however, were not known.” The expression “not known” is too strong. Please downplay this.*

**We made this change.**

*4) The authors showed that autophagic induction was enhanced by BMP signaling in a daf-2 mutant background. However, the description of autophagy regulation is lacking in the introduction part, and the reason why rpl-43 mutant was used in S1 Fig should be mentioned in the Results.*

**We preferred not to make the introduction longer and to keep the background for autophagy experiments where the reader will need it. We explained the effect of *rpl-43* mutation in the Results section (p. 14).**

*5) Add citation to the first sentence on page 4, “Disruptions to insulin balance, such as insulin resistance, can lead to multiple metabolic disorders and diseases, including obesity and type II diabetes.”*

**We added a citation (p. 3).**

*6) Several sentences are ambiguous and hard to understand because they do not include any directions that the authors want to claim. For example, in page 6, the authors wrote “As previously published, we saw a significant decrease in sma-3 animals by ~42% (p<0.0001).” I think the authors wanted to say, “As previously published, we saw a significant decrease in lipid accumulation in sma-3 animals by ~42% (p < 0.0001).”*

**We made this clarification.**

*7) Scale bars for all images in Figures are missing.*

**In each panel, images are taken at the same magnification and relevant anatomical features are noted.**

*8) In Figure 5, it will be helpful to add representative images of the animals stained with Oil Red O.*
**We did not want to increase the size of the manuscript further.**

**Reviewer #2:**

*In this manuscript, Clark et al. describe genetic interactions between the DBL-1/BMP and DAF‑2/IIS pathways, extending previous work by implicating INS-4 and describing intriguing differential effects on the SKN-1 and DAF-16 transcription factors. The new data are interesting and add to our understanding of how these pathways influence each other. Unfortunately, however, the work is broader than it is deep, and for the most part these interesting observations are not pursued mechanistically to a sufficiently illuminating extent. In addition, the novelty is limited by the prior work done in these areas. While the paper reports a number of interesting findings, it would be a much stronger candidate for PLoS genetics if it developed at least some of these areas more thoroughly mechanistically.*

**We have considered this point and have deepened our focus on the previously unexplored relationship between insulin-like peptide INS-4 and downstream transcription factors DAF-16/FoxO and SKN-1/Nrf in lipid homeostasis. We have now added data in (Figure 3B,C) that show that the loss of *daf-16* blocks the high-fat phenotype of *ins-4* mutants and that DAF-16::GFP nuclear localization in *daf-2* RNAi animals is sensitive in *ins-4* overexpression.**

Specific comments:

**In responding to these specific comments, it was necessary to choose an area of focus to develop and the other suggestions, though interesting, are beyond the scope of the paper as indicated below.**

*1. In Fig. 2 there is no WT control - even though it is well-known that decreased insulin-receptor signaling promotes autophagy a full experiment still needed to put the data in context. The image quality also leaves something to be desired. Most importantly, the authors need to acknowledge that they are looking at steady-state LGG-1, not the rate of autophagy. More recent approaches by the Hansen lab have measured autophagic flux and looked in additional issues. This may not necessarily need to be addressed comprehensively, but must be discussed in the context of caveats.*

**We added a caveat in the Results section that describes how we have analyzed steady-state levels of LGG-1 foci (p. 15).**

*2. Can the authors exclude a role for INS-4 in neurons? Are there physiological conditions through which sma-3 regulates ins-4? Can restoration of sma-3 rescue the epistasis phenotype (helpful, not essential). Given the central importance of INS-4, going deeper mechanistically would strengthen the work.*

**INS-4 function in neurons is sufficient to regulate fat stores (Zheng et al., JBC 2018). What our work shows for the first time is that *ins-4* expression is regulated by SMA-3 in the hypodermis, which is also the tissue in which SMA-3 acts to regulate fat storage.**

*3. Are fat stores affected independently of reproduction? Wang/Ruvkun have shown that germline stem cell ablation (decreased reproduction) increases fat deposition.*

**We added new experiments (S1 Fig) showing that, in addition to regulating fat storage in L4 larvae, SMA-3 also regulates fat storage in reproductive adults and in adults in which progeny production is blocked by FUDR. Thus, there is not a strong correlation between reproductive activity and the *sma-3* low-fat phenotype.**

*4. In many places where only a single mutant is used, analysis of more pathway components would have strengthened the work. At the same time, lon-2 appears out of the blue. Diagrams of these pathways would be helpful for non-aficionados.*

**We added a description of LON-2 in the Introduction.**

*5. Many findings that are interesting are left hanging as loose ends. For example, how might DAF-16::GFP increase fat accumulation? How might DAF-16 but not SKN-1 nuclear localization be affected by dbl-1? These may be difficult questions, but they illustrate the problem of depth in the work.*

**We agree with the reviewer that these are very interesting questions, but they are beyond the scope of this manuscript at this time.**

*6. The dauer decision is influenced by temperature independently of IIS and TGFb signaling (see original Riddle papers). In Table 1, how can the authors know whether the BMP mutants affect dauer entry by influencing IIS rather than sensitivity to temperature (or a different variable)? This is later backed up by DAF-16 data, but the logic is not developed.*

**We have now reorganized the presentation of data to improve the logic flow.**

*7. Here and there important results are not referenced. Two examples can be found in the first paragraph of the introduction, where the authors do not cite work from their own lab! Another example is the description of dauer regulation on page 7. Overall the introduction is diffuse and jumps around, and would be improved by tightening. The entire paper would be improved if it were shorter and more tightly focused.*

**We added references and re-organized the paper.**
