# Supplementary material for: BMP pathway regulation of insulin signaling components promotes lipid storage in Caenorhabditis elegans
Source: PLoS Genet. 2021 Oct 11;17(10):e1009836. doi: 10.1371/journal.pgen.1009836 (PMC8530300; doi:10.1371/journal.pgen.1009836)
Supplement: Attachment — Submitted filename: Response to Reviewers PGENETICS.docx [file pgen.1009836.s004.docx]

**Response to Reviewers *PGENETICS-D-21-01120***

We thank the reviewers for their thoughtful reviews and helpful suggestions for improving the clarity of our manuscript. We made the following changes in response to specific comments of the reviewers.

*Reviewer #1: Comments to PGENETICS-D-21-01120

I have read the reviewer responses and the manuscript. To me, the previous reviewers’ requests were adequately addressed. I agree with the previous reviewer suggestion to focus on the most novel findings to gain more mechanistically insights. I also agree with the choice of the authors’ to focus on ins-4 and the interaction with BMP signaling.

I only have two concerns that can be addressed by text changes.

1) Clarification of the genetic model for fat accumulation. Figure 1: Loss of sma-3 increases ins-4 expression levels, suggesting that sma-3 is upstream of ins-4. In the analysis for fat accumulation, loss of sma-3 leads to lower fat levels vs loss of ins-4 mildly increases fat levels, hence opposite phenotypes making it a good system to determine epistasis. One would expect if fat reduction of loss of sma-3 would act through ins-4 (as suggested in the model), then the double mutant of sma-3;ins-4 should phenotypically look like ins-4 (mild increase in fat) but shows the opposite (ie, lower fat) phenocopying sma-3. This suggests that the fat reduction of loss of sma-3 is independent of ins-4. A clarification of this finding and how it fits the model would be great.*

**We have clarified this expectation for epistasis in the text, and how we interpret the result to suggest that INS-4 is not the only ILP acting downstream of SMA-3. On page 10, we state: “If INS-4 were the only ILP acting downstream of SMA-3, we would expect the ins-4 high-fat phenotype to be epistatic to the sma-3 low-fat phenotype, similarly to the daf-2/InsR high-fat phenotype as previously described (Clark et al., 2018).”**

*2) When I was reading this manuscript, I kept asking myself, how does loss of sma-3 lead to such a strong loss of fat levels. It is a pretty striking phenotype with 50-75% fat loss compared to WT when looking at fig 1 and fig S1. Could the higher or reduced fat accumulation be simply due to changes in feeding rates? Recently, daf-2/daf-16 signaling has been shown to regulated feeding rates (PMID: 30905669). Could the observed changes in fat levels and autophagy be simply due to different feeding rates in dbl-1/sma-3 and ins-4? Would smaller body size not also correlate with lower feeding rates? I understand coming into a revised manuscript as a new reviewer, it is not fair to ask for addition experiments (besides control experiments). Thus, I do not ask for the experiments but rather a discussion as possibility to think about and for perhaps future directions. May be the authors already know good arguments of why feeding rates is not a likely explanation of the observed phenotypes.*

**We agree with the reviewer and wondered whether reduced feeding could contribute to lower fat accumulation and small body size. We previously addressed this potential mechanism by measuring the pumping rates in *dbl-1* and *sma-3* mutants and found, however, that they were slightly elevated (*dbl-1)* or not significantly different (*sma-3)* from wild type (Clark et al., 2018), suggesting that altered feeding rate is not a major contributor to the fat accumulation. We also showed that body size and lipid accumulation are separable phenotypes. We have added a phrase in the Results to clarify the pharyngeal pumping result. On page 9, we state: “In previous work, we showed that SMA-3/Smad activity in the hypodermis (epidermis) regulates fat accumulation in the *C. elegans* intestine, and that SMA-3/Smad regulation of fat stores depends on the DAF-2 Insulin Receptor (InsR) and is not associated with altered pharyngeal pumping that would modulate feeding rate (Clark et al. 2018).”**

*Reviewer #2: In this manuscript, Clark et al., identify an insulin-like peptide, ins-4, as a mediator of BMP and daf-2/IIS pathway interactions to regulate lipid metabolism. The data presented are intriguing and will be of interest to the C. elegans community. However, a couple of key experiments are missing that would strengthen the authors’ most novel claim that BMP signaling regulates ins-4 in the hypodermis to regulate fat metabolism.

The authors argue that sma-3 in the hypodermis regulates expression of ins-4, and state that ins-4 is “highly and specifically increased in the hypodermis in the sma-3 mutant. However, the only piece of evidence that ins-4 levels are increased in the hypodermis is based on fluorescent reporter images. It is not clear whether ins-4::GFP fluorescence is also increased in neurons based on the images provided. While I appreciate that they confirmed that ins-4 mRNA is increased in sma-3 mutants, this is done from whole animal lysate, so it is not appropriate to make any claims of tissue specificity. Because this cell non-autonomous communication contributes to the novelty of the manuscript, I think the authors should perform a more direct experiment to determine if INS-4 is originating from hypodermis to regulate lipid metabolism. For example, does tissue specific overexpression of sma-3 in hypodermis (strain published in the author’s previous paper) in the sma-3 mutant background reduce ins-4:GFP in the hypodermis?*

**We appreciate this reviewer’s concern, however we didn’t observe any increase in *ins‑4p::gfp* expression in neurons in *sma-3* mutants. Because we didn’t quantify the expression in neurons, though, we have now deleted the phrase “and specifically” from the text where we had stated “highly and specifically” in the Discussion. We further clarified in the text that *sma-3* is expressed in pharynx, intestine, and hypodermis, but not observed in neurons. Therefore, SMA-3 is not expected to regulate *ins-4* in neurons.**

*In Figure 3C, it is not clear if the ins-4 overexpression is hypoderm-specific. This manuscript would be significantly strengthened if the authors provide data that ins-4 overexpression in the hypodermis induces daf-16 nuclear localization in the intestine and affects lipid metabolism. Ins-4 loss-of-function experiments via ins-4 hypoderm-specific RNAi, for example, would also be informative.*

**The overexpression studies presented use the native promoter of *ins-4* and not a tissue-specific promoter. As a secreted factor, INS-4/ILP can act to regulate lipid metabolism when expressed in different tissues. For example, INS-4 expression in neurons is sufficient to regulate fat stores (Zheng et al., JBC 2018). Thus, we would expect that overexpression from the hypodermis would also show an effect. Our work demonstrates that the native *ins-4* promoter is de-repressed in the hypodermis in response to genetic loss of SMA-3/Smad.**
